# Cognitive Flexibility and Emotion Regulation in Eating Disorder Patients with Comorbid Generalized Anxiety and Posttraumatic Stress Symptoms

**DOI:** 10.21203/rs.3.rs-4326208/v1

**Published:** 2024-04-29

**Authors:** Connor J Thompson, Caitlin A Martin-Wagar

**Affiliations:** University of Montana

**Keywords:** eating disorders, comorbidity, emotion regulation, cognitive flexibility, generalized anxiety disorder, posttraumatic stress disorder

## Abstract

Research has found that difficulties in emotion regulation negatively impact mental health, whereas cognitive flexibility may promote stress resilience and positive mental health. Little is known about cognitive flexibility and emotion regulation in people with comorbid eating disorder (ED) and anxiety and stress disorders. A transdiagnostic ED population (N=227) at an outpatient ED treatment facility completed several self-report instruments that measured cognitive flexibility, emotion regulation difficulties, posttraumatic stress disorder (PTSD) symptoms, and generalized anxiety disorder (GAD) symptoms upon admission. We investigated cognitive flexibility and emotion regulation differences for those with an ED without comorbidity and those with various combinations of comorbidity. In a one-way between-groups ANOVA, we investigated differences in cognitive flexibility for those with GAD, PTSD, neither, and both comorbidities. We found a statistically significant difference between these groups, with mean cognitive flexibility inventory scores being the lowest in the group with both comorbidities. However, when controlling for emotion regulation, a one-way between-groups ANCOVA indicated no significant differences in cognitive flexibility between comorbidity groups F(3,222)=1.20,p=.31 *Partial* $\eta^{2}\;=\;.02$. Though self-reported cognitive flexibility levels differ among ED patients with and without comorbidities, it appears that these differences are better explained by emotion regulation. Therefore, addressing emotion regulation early in treatment for all individuals with EDs, regardless of comorbidity. Further research is needed to understand the impact of treating emotion regulation on ED treatment engagement, dropout, and effectiveness.

## Introduction

Individuals with an eating disorder (ED) are highly likely to have a psychiatric comorbidity. Those with anorexia nervosa (AN) are almost four times more likely to receive a secondary psychiatric diagnosis than those without AN (Momen et al., 2023). Similarly, those who have received a diagnosis of a psychiatric disorder are over four times more likely to develop AN than those without a psychiatric diagnosis. For EDs in general, some of the most common psychiatric comorbidities are anxiety disorders and posttraumatic stress disorder (PTSD; Hambleton et al., 2022). Although PTSD and ED comorbidities have been well-documented, the relationship is not well understood, and no evidence-based treatment for these concurrent disorders is currently available (Mitchell et al., 2021). While the directionality of these comorbid diagnoses is not well understood, it might be that for some, ED symptoms serve as a coping strategy to help mitigate symptoms of generalized anxiety disorder (GAD) and PTSD (Mitchell & Wolf, 2016). Avoidance, a core symptom of PTSD and many anxiety disorders, may also be involved with these comorbidities (Trottier et al., 2016).

Comorbidity in psychiatric disorders is quite common, as many individuals with one psychiatric illness will experience a second psychiatric illness throughout their lifespan (McGrath et al., 2020). A 2012 study conducted by Kessler and colleagues found that a significant number of adolescents with an anxiety disorder developed a later-onset disorder(s), positing that anxiety disorders should be targets for early intervention for the prevention of comorbid psychiatric illness. Multiple comorbidities are disadvantageous for several reasons, such as reduced quality of life, worsened physical and mental health, and increased mortality (Salisbury et al., 2018). Though adverse outcomes are abundantly associated with many comorbid illnesses, the most common co-occurring mental illnesses are GAD, PTSD, and depression (Price et al., 2020).

Inflexible behavior is also exhibited in GAD, where experiential avoidance (avoidance of emotions or internal experiences) is much more prevalent than in non-clinical groups (Lee et al., 2010). Rigid behaviors such as calorie restriction, compensatory behaviors, and rules around eating are also inflexible behaviors commonly seen in those with EDs. Likewise, avoidance of others, overworking to avoid triggers, and substance abuse to avoid negative internal experiences are all inflexible behaviors frequently displayed by those with PTSD (U.S. Department of Veterans Affairs, 2023). Additionally, individuals with PTSD frequently experience rigid and maladaptive thought patterns, including negative beliefs about the self, avoidance of traumatic memories, and overgeneralized feelings of ongoing threats (Thompson-Hollands et al., 2017; Ehlers & Clark, 2000; U.S. Department of Veterans Affairs, 2023).

Cognitive flexibility encapsulates an individual’s ability to adjust their thinking and broaden their awareness of choices when met with unexpected or challenging environmental demands (Roberts et al., 2011). Though avoidance and coping functions of eating disorder symptomology may help to partially explain co-occurring GAD or PTSD with EDs, a deficiency in cognitive flexibility (CF) has also been shown in those with EDs, GAD, or PTSD (Grant & Chamberlain, 2023; Tchanturia et al., 2012). Cognitive inflexibility has also been presented as a possible contributor to those with social anxiety disorder and a co-occurring eating disorder (Arlt et al., 2016). Cognitive inflexibility has not been examined extensively, however, in those with EDs and comorbid GAD, PTSD, or both. By measuring cognitive flexibility in those with an ED and comorbid GAD, PTSD, or both, we hope to expand upon the literature surrounding the role of cognitive flexibility in adverse symptoms of mental illness. Furthermore, if ED patients with these comorbidities experience lower levels of cognitive flexibility than those without a comorbid diagnosis, it may be key to identify comorbidities upon intake and address cognitive inflexibility, if present.

In some instances, eating pathology development and maintenance can result, in part, from general difficulties in regulating emotions and behavior in combination with other biopsychosocial factors (Leppanen et al., 2022). Across the spectrum of eating disorders, individuals often have difficulty accepting emotions. For instance, Monell and colleagues (2018) showed that patients with AN had a greater deficiency in emotion regulation than the comparison group (healthy students). However, ED symptoms were found to be associated with emotion dysregulation regardless in both the AN and non-clinical comparison groups. Another recent study found an interaction between emotion regulation and cognitive flexibility to be associated with more problematic eating pathology (Dann et al., 2022). When considering those with a comorbid PTSD and ED diagnosis, emotion regulation deficiencies (e.g., outbursts of anger, avoidance, or impulsive behavior) are quite common (Trottier & MacDonald, 2017). Similarly, emotion regulation difficulties are prominent in those with GAD, as generalized anxiety often coincides with emotional and cognitive avoidance (Borkovec et al., 2004). Worry (a primary characteristic of GAD) is often used as a means of emotional and experiential avoidance (e.g., utilizing worry about an external life event as a means of avoiding distressing or intense emotions that are experienced internally), further exhibiting emotion regulation difficulties in those with GAD (Hayes et al., 1996; Oathes et al., 2011; & Roemer et al., 2005). Importantly, emotion regulation difficulties, such as impulsivity, have been found to predict premature ED treatment dropout (Fassino et al., 2009). With ED treatment dropout being particularly high, identifying who might be at higher risk for these difficulties upon admission is warranted and may provide insight into who is at higher risk for dropout.

In the present study, to better illuminate patients at risk for premature dropout and recovery barriers, we first aimed to compare levels of self-reported cognitive flexibility between those with an ED (no GAD comorbidity) and those with comorbid ED and GAD. We hypothesized that GAD comorbidity would be related to higher levels of cognitive inflexibility, when compared to an ED sample without GAD comorbidity. Our second aim was to compare levels of self-reported cognitive flexibility between those with an ED and no PTSD comorbidity to those with an ED and comorbid PTSD. We hypothesized that the comorbid group would report lower levels of total cognitive flexibility than the non-PTSD sample. The third aim of this study was to investigate whether a compounding effect is present in those with both GAD and PTSD comorbidities. We hypothesized that ED patients with both comorbidities would report lower levels of cognitive flexibility than those with GAD-only or PTSD-only comorbidity. The fourth aim of this study was to examine whether emotion regulation difficulties would better explain these different comorbidity groups and their relationships with cognitive flexibility.

## Methods

### Participants and Procedures

Participants were adults seeking treatment within an eating disorder specialty clinic in the Midwestern United States (N=227). All participants completed measures as part of the intake process at the clinic and were asked for consent for their measures to be used for research purposes. They were informed that their relationships with treatment, providers, and the clinic would not be impacted by their decision to participate in research. Participants were also asked to consent for the research team to view their electronic medical records to confirm ED diagnoses and anthropometric data. During the intake procedures, participants were assessed for the presence of an eating disorder. Intake clinicians were licensed mental health providers who used diagnostic criteria from the Diagnostic and Statistical Manual of Mental Disorders − 5th Edition (American Psychological Association, 2013) and who specialized in eating disorders. All study procedures were approved by a university Institution Review Board in the Midwestern United States.

Participant age ranged from 18 to 67 (M=31.91), and of the body mass indexes available from chart review (n=172), BMI ranged from 11.20–60.10 (M=25.39) in this transdiagnostic eating disorder sample. Additional participant demographics can be found in Table 1.

### Measures

#### Posttraumatic stress disorder symptoms.

Posttraumatic stress disorder symptoms were measured using the Posttraumatic Stress Disorder Check List for the DSM-5 (PCL-5; Blevins et al., 2015). Participants received this measure only if they endorsed having experienced a traumatic event on the trauma history screen (THS). The PCL-5 consists of 20 items, measuring the 20 DSM-5 symptoms of posttraumatic stress disorder (PTSD). Each item of the PCL-5 is scored on a 5-point Likert scale, with participants rating how much they have been bothered by each of these symptoms in the past month, from 0 (not at all) to 4 (extremely). An overall score for the PCL-5 can be calculated by summing the scores on all 20 items (range 0–80), with a clinical cutoff score of 31 (Bovin et al., 2016). Upon its initial development, Blevins and colleagues (2015) found that PCL-5 scores exhibited strong internal consistency (α=.94), test-retest reliability (r=.82), and convergent (r=.74to.85) and discriminant (r=.31to.60) validity. In a network analysis of posttraumatic stress and eating disorder symptoms in a sample of adults, internal consistency was excellent (α=.95; Liebman et al., 2021). McDonald’s omega for the PCL-5 in this study was found to be 0.96, indicating good reliability (Hayes & Coutts, 2020).

#### Generalized Anxiety Disorder.

Generalized anxiety symptoms were measured using the Generalized Anxiety Disorder 7-item Scale (GAD-7; Spitzer et al., 2006). The measure consists of 7 items that measure for symptoms of anxiety (similar to the generalized anxiety disorder diagnostic criteria described in the DSM). The GAD-7 then asks participants to rate how often they have been bothered by these symptoms over the past two weeks (e.g., “Feeling nervous, anxious, or on edge”) on a 4-point Likert scale, ranging from 0 (Not at all) to 3 (Nearly every day). An overall score on the GAD-7 can be calculated by summing item scores of the 7 responses. The clinical cut-off score is 10, and the GAD-7 is regarded as a useful screening tool for GAD (Kroenke et al., 2007). The current study found good reliability on the GAD-7, with a McDonald’s omega value of 0.92.

#### Cognitive Flexibility.

Levels of cognitive flexibility were measured using the Cognitive Flexibility Inventory (CFI; Dennis & Vander Wal, 2010). The CFI measures one’s ability to generate multiple alternative solutions to different situations and one’s tendency to perceive difficult situations as controllable (Johnco et al., 2014). When taking the CFI, participants are asked to rate how much they agree with 20 statements, scoring items on a 7-point Likert scale, ranging from 1 (strongly disagree) to 7 (strongly agree). A total score (ranging from 20 to 140) can be calculated by summing all 20 items, with higher scores indicating higher cognitive flexibility. Total CFI score had good reliability in this study, with a McDonald’s omega value of 0.91.

#### Emotion Regulation.

Self-reported emotion regulation skills were measured using the Difficulties in Emotion Regulation Scale (DERS; Gratz & Roemer, 2004). The DERS has 36 items and asks participants to indicate how often these statements apply to them (e.g., “I experience my emotions as overwhelming and out of control”) on a 5-point Likert scale, ranging from 1 (almost never, 0–10%) to 5 (almost always, 91–100%). Higher scores indicate greater problems with emotion regulation (Gratz & Roemer, 2004). The DERS had good reliability in this study, with McDonald’s omega being 0.95.

### Analytic Plan

IBM SPSS Statistics 28.0 was used to examine the study hypotheses. First, we computed total GAD-7 and PCL-5 scores. We were then able to make probable comorbidity groups in each variable based on the clinical cutoff for the GAD-7 and the PCL-5 (scores of 10 and 31, respectively; Spitzer et al., 2006; Blevins et al., 2015). In doing so, we were able to compare groups that had no GAD and PTSD comorbidity, a probable GAD comorbidity, a probable PTSD comorbidity, or both comorbidities on cognitive flexibility and emotion regulation.

Next, two independent samples t-tests were used to examine whether cognitive flexibility differences were present between the GAD and no GAD comorbidity groups or the PTSD and no PTSD comorbidity groups. Afterward, we conducted a one-way between-groups analysis of variance (ANOVA) to examine differences in cognitive flexibility among four groups of individuals with EDs: the GAD-only comorbidity, PTSD-only comorbidity, both comorbidities, and no GAD or PTSD comorbidities. Previous research has proposed that cognitive flexibility is an underlying mechanism of effective emotion regulation (Genet & Siemer, 2011; Oschsner & Gross, 2007), which prompted a one-way between-groups analysis of covariance (ANCOVA) that controlled for emotion regulation. This allowed us to examine if self-reported difficulties with emotion regulation might help to explain potential differences in cognitive flexibility amongst ED patients with and without comorbid anxiety disorders.

## Results

Using scale-level analyses of missing data, we found no missing items across the CFI, DERS, PCL-5, or GAD-7. Thus, no adjustments to the data set were needed to account for missing data.

### Clinical Cutoffs in the Outpatient ED Sample

In the transdiagnostic ED outpatient sample (N=227), 25.1% met the clinical cutoff for only GAD, 4.0% met the clinical cutoff for only PTSD, and 41.9% met clinical cutoffs for both PTSD and GAD on the PCL-5 and the GAD-7, respectively. 29.1% of the ED patients did not meet clinical cutoffs for either GAD or PTSD.

### Differences Between GAD, PTSD, and Non-Comorbidity ED Patients

In an independent samples t-test, comparing the GAD group to the non-GAD group, mean CFI levels were significantly lower in those with GAD comorbidity (M=85.28,SD=18.80) than in those without GAD comorbidity (M=102.27,SD=14.83);t(225)=6.84,p<0.001,d=.97 (large effect), 95% CI [.674, 1.26].

In the second t-test, comparing the PTSD group to the non-PTSD group, CFI global scores for those with PTSD comorbidity (M=83.31,SD=18.60) were significantly lower than those without PTSD comorbidity (M=97.30,SD=17.54);t(225)=5.83,p<.001,d=.78 (large effect), 95% CI [.505, 1.046].

### Comparing CFI Scores Across Comorbidity Groups

In the one-way between-groups ANOVA, we investigated differences in self-reported CFI global scores for the GAD and PTSD comorbidity group, compared to the GAD-only group, PTSD-only group, or no comorbidity group. Levene’s test of equality of error variances was significant (p=.047), indicating the data did not meet the assumption of homogeneity of variances. As such, Welch statistic was used to examine differences in CFI scores amongst the four comorbidity groups, F(3,52.486)=31.256,p<.001, Partial $\eta^{2}\;=\;.22$ (large effect).

Tukey’s HSD Test for multiple comparisons indicated that the mean CFI score of those with no comorbidities (M=101.85,SD=15.64) was significantly higher than those in the GAD-only group (M=92.04,SD=18.26,p=.009, 95% C.I. = [1.80, 17.83]), and those with both comorbidities (M=81.22,SD=18.30,p<.001, 95% C.I. = [13.53, 27.73]). Tukey’s HSD also indicated that mean CFI scores were lower in those with both comorbidities than in both the GAD-only (p=.001, 95% C.I.=[−18.24,−3.39]) and PTSD-only groups (M=105.33,SD=6.02,p<.001, 95% C.I.=[−39.57,−8.65]). There were no significant differences between the no comorbidity and PTSD-only groups (p=.940) or between the GAD-only and PTSD-only groups (p=.136).

Next, we examined differences in CFI global scores for the GAD-only group, the PTSD-only group, the no comorbidity group, and the group that had both comorbidities, using self-reported emotion regulation abilities from the DERS as a covariate in a one-way between-groups ANCOVA. No significant differences in CFI scores were found between the groups after controlling for DERS score, F(3,222)=1.20,p=.31 *Partial* $\eta^{2}\;=\;.02$. We found a significant relationship between the CFI and DERS scores, *Partial* $\eta^{2}\;=\;.27$.

## Discussion

In line with our hypothesis on cognitive flexibility differences between the GAD and non-GAD comorbidity groups, total cognitive flexibility was significantly lower in individuals with a GAD comorbidity compared to those without GAD comorbidity. Our hypothesis regarding cognitive flexibility differences between the PTSD and non-PTSD comorbidity groups was supported, as total cognitive flexibility was significantly lower in those with comorbid PTSD than in those with no PTSD comorbidity.

Supporting our hypothesis on the compounded effect of having both GAD and PTSD comorbidities, total cognitive flexibility was significantly lower in individuals with both GAD and PTSD than in individuals with singular GAD or PTSD comorbidity or no comorbidities. This finding is in line with previous research on the negative implications of comorbid illness and PTSD, which can exacerbate one another, leading to further complications in both (e.g., avoidance of feared stimuli, negative coping strategies; Price et al., 2019; van Dam et al., 2013). Further, these findings build upon past research on cognitive flexibility deficiencies in those with EDs, GAD, or PTSD (Grant & Chamberlain, 2023; Tchanturia et al., 2012), elaborating on the intersection of these illnesses and the relationships that these various comorbidities have with cognitive flexibility. However, our later findings seem to suggest that these differences can be attributed to differing levels of emotion regulation ability.

These findings are somewhat consistent with prior research on emotion regulation deficiencies in those with PTSD and an ED (Trottier & MacDonald, 2017), showing that the comorbidity of the two often co-occurs with difficulty regulating one’s emotions. Similarly, these findings coincide with previous findings on emotion regulation difficulties in those with GAD, as generalized anxiety is often accompanied by avoidance of negative experiences (Borkovec et al., 2004). For instance, worry (a central characteristic of GAD) is often used as a means of emotional and experiential avoidance (e.g., utilizing worry about an external life event as a means of avoiding distressing or intense emotions that are experienced internally), further exhibiting emotion regulation difficulties in those with GAD (Hayes et al., 1996; Oathes et al., 2011; & Roemer et al., 2005). Emotion regulation deficiencies are of great importance across mental health treatment settings, as impulsivity based on emotional reactivity is a predictor of premature treatment dropout (Fassino et al., 2009). While those with comorbidity do have lower cognitive flexibility, it appears the individual’s emotion regulation abilities are more directly related to cognitive flexibility (and not comorbidity status). Thus, measuring emotion regulation at intake may be one of the most important variables, rather than relying on comorbid diagnoses alone.

Dann and colleagues (2022) measured flexibility pertaining to eating pathology, finding that flexibility was an independent predictor of eating pathology (i.e., cognitions and compensatory behaviors). In our sample, we found cognitive flexibility to be insignificant in the prevalence of potential stress and anxiety comorbidity in an ED sample, after controlling for difficulties in regulating emotions. However, we did not examine these variables in relation to ED pathology but among comorbidity groups. Additional methodologies are needed to fully examine the relationships between ED symptoms, comorbidity, emotion regulation, and cognitive flexibility. Future clinical research should focus on further attempts to tease out how these factors interact, and should examine the role of cognitive flexibility in the development and treatment of eating disorders and comorbid illnesses.

### Limitations

Though this project contributes new knowledge about comorbid symptoms, cognitive flexibility, and emotion regulation in a clinical ED sample, several limitations exist. The first is that participants completed self-report measures of PTSD and GAD without receiving a formal diagnosis from a mental health clinician. Thus, our findings apply best to understanding comorbidity symptoms, and we cannot be sure these findings will remain in a sample that also had a clinical interview to determine all relevant comorbid diagnoses. We did use well-established clinical cut-offs for the GAD-7 and PCL-5, which have an 82% specificity and 96% specificity, respectively (Spitzer et al., 2006; Blevins et al., 2015). As such, we feel our findings provide relevant information about how comorbid GAD and PTSD symptoms function in those with EDs.

Another limitation of our sample is that it is comprised of a treatment-seeking population of those with EDs. While using a clinical sample is a strength, it is also a limitation in this case because insurance-based treatment centers like the one used in this study tend to have a more homogenous population with little racial and ethnic diversity due to inequitable access to insurance and other barriers to receiving an ED diagnosis. As such, other studies should examine the variables in other samples, such as treatment samples from grant-funded studies that do not require insurance.

## Conclusions

These findings indicate the importance of emotion regulation on cognitive flexibility, regardless of comorbidity. Although emotion regulation seems to be the driver of these differences in cognitive flexibility rather than comorbidity itself, it is not to say that cognitive flexibility has no role in ED patients with comorbidity. Instead, future research should examine the best ways to address emotion regulation early in treatment and its potential for impacting ED treatment engagement, dropout, and effectiveness. Existing ED treatments are already insufficient in helping a large proportion of those with EDs reach and maintain recovery (Linardon, 2018). Therefore, addressing emotion regulation early in treatment for all individuals with EDs, regardless of comorbidity, is a recommended avenue for future clinical research aimed at improving ED treatment outcomes.

## Figures and Tables

**Table 1:** Participant Demographics (*N* = 227)

	*n*	%

Race/Ethnicity		
White/European American	213	93.8
Black/African American	4	1.8
Asian American	2	0.9
Hispanic/Latinx	4	1.8
Native American	1	0.4
Middle Eastern	1	0.4
Bi/Multi-Racial	2	0.9

Sexual Orientation		
Heterosexual/Straight	199	87.7
Lesbian/Gay	5	2.2
Bisexual/Pansexual	15	6.6
Asexual	1	0.4
Demisexual	1	0.4
Questioning/Curious/Uncertain	4	1.8
Did not respond	2	0.9

Annual Household Income		
Less than 10,000	35	15.4
10,000–49,000	75	33.0
50,000–89,000	51	22.5
90,000–149,000	34	15.0
150,000 or more	24	10.6
Did not respond	8	3.5

Eating Disorder Diagnosis		
Anorexia Nervosa	74	32.6
Bulimia Nervosa	28	12.3
Binge Eating Disorder	54	23.8
Other Specified Eating Disorders	71	31.3

**Table 2:** Descriptive Statistics on GAD and Non-GAD Groups (*N* = 227)

	Total Sample		GAD		Non-GAD	
*Variable*	*M*	*SD*	*M*	*SD*	*M*	*SD*
*CFI*	90.89	19.30	85.28	18.80	102.27	14.83

CFI = Cognitive Flexibility Inventory; GAD = Generalized Anxiety Disorder

**Table 3:** Descriptive Statistics on PTSD and Non-PTSD Groups (N = 227)

	Total Sample		PTSD		Non-PTSD	
*Variable*	*M*	*SD*	*M*	*SD*	*M*	*SD*
*CFI*	90.89	19.30	83.31	18.60	97.30	17.54

CFI = Cognitive Flexibility Inventory; PTSD = Posttraumatic Stress Disorder

## Data Availability

The datasets generated and/or analyzed during the current study are available in the Open Science Framework repository, https://osf.io/ksx8u.

## Clinical Implications

- Eating disorder patients with comorbidities had the lowest cognitive flexibility.
- When accounting for emotion regulation, group differences were no longer present.
- Addressing emotion regulation early in eating disorder treatment is recommended.
